# Retraction: Investigation of anticorrosive, antibacterial and *in vitro* biological properties of a sulphonated poly(etheretherketone)/strontium, cerium co-substituted hydroxyapatite composite coating developed on surface treated surgical grade stainless steel for orthopedic applications

**DOI:** 10.1039/d3ra90028b

**Published:** 2023-03-29

**Authors:** D. Rajeswari, D. Gopi, S. Ramya, L. Kavitha

**Affiliations:** a Department of Physics, Periyar University Salem 636 011 Tamilnadu India; b Department of Chemistry, Periyar University Salem 636 011 Tamilnadu India dhanaraj_gopi@yahoo.com +91-427-2345124 +91-427-2345766; c Centre for Nanoscience and Nanotechnology, Periyar University Salem 636 011 Taminadu India; d Department of Physics, School of Basic and Applied Sciences, Central University of Tamilnadu Thiruvarur 610 101 India

## Abstract

Retraction of ‘Investigation of anticorrosive, antibacterial and *in vitro* biological properties of a sulphonated poly(etheretherketone)/strontium, cerium co-substituted hydroxyapatite composite coating developed on surface treated surgical grade stainless steel for orthopedic applications’ by D. Rajeswari *et al.*, *RSC Adv.*, 2014, **4**, 61525–61536. https://doi.org/10.1039/C4RA12207K.

The Royal Society of Chemistry, with the agreement of the authors, hereby wholly retracts this *RSC Advances* article due to concerns with the reliability of the data in the published article.

The HRSEM image in Fig. 4c represents a scaled version of part of the image presented in Fig. 4e. However, both images are presented with the same scale bar and represent different samples.

Repeating fragments can be observed in the images in Fig. 6a–f, indicating that the images have been manipulated.

The optical images in Fig. 8a–c have been duplicated in other publications. The panel in Fig. 8a has been duplicated as Fig. 9a in ref. 1, and as Fig. 6a and c in ref. 2. The panel in Fig. b has been duplicated as Fig. 9b in ref. 1, and as Fig. 6e in ref. 2. The panel in Fig. 8c has been duplicated as Fig. 9c in ref. 1.

The authors informed the editor that the characterization of the original samples was outsourced, and they do not have the original raw data for the published results.

Given the significance of the concerns about the validity of the data, and the lack of raw data, the findings presented in this paper are not reliable.

Signed: D. Rajeswari, D. Gopi, S. Ramya and L. Kavitha

Date: 16th March 2023

Retraction endorsed by Laura Fisher, Executive Editor, *RSC Advances*

## Supplementary Material
